# Effect of Timing of Initial Cataract Surgery, Compliance to Amblyopia Therapy on Outcomes of Secondary Intraocular Lens Implantation in Chinese Children: A Retrospective Case Series

**DOI:** 10.1155/2018/2909024

**Published:** 2018-03-22

**Authors:** Liuyang Li, Yan Wang, Caihong Xue

**Affiliations:** ^1^Clinical College of Ophthalmology, Tianjin Medical University, Tianjin, China; ^2^Tianjin Eye Hospital and Eye Institute, Tianjin Key Lab of Ophthalmology and Visual Science, Tianjin, China

## Abstract

**Purpose:**

As a secondary analysis, we reassess the association of initial congenital cataract surgery times, compliance to amblyopia therapy, and visual outcomes for a long-term follow-up in a secondary IOL implantation.

**Methods:**

Retrospective review of records of all infants with congenital cataracts who underwent secondary IOL implantation in the Eye and ENT Hospital of Fudan University from January 1, 2001, to December 31, 2007, and the minimum follow-up period was 5 years. Multiple regression analysis was used and the possible confounding factors were also analyzed to assess the effect on visual outcome.

**Results:**

A total of 110 patients (male: 59.1%) were included. The median (min–max) age at cataract extraction and IOL implantation was 7.5 (3.0–15.0) and 35.0 (22.0–184.0) months, respectively, and the average follow-up period was 99.3 ± 23.6 months. The median (min–max) BCVA at final follow-up was 0.20 (0.01–1.00). Compliance to amblyopia therapy was none, poor, and good in 21.8%, 24.5%, and 53.6%, respectively. Postoperative BCVA [logMAR, median (min–max) 0.70 (0.00–2.00)] linearly decreased with increasing cataract extraction time (per month) (*β* = 0.04, 95% CI: 0.03–0.06, *p* < 0.0001) in multivariable models with laterality and compliance to amblyopia therapy adjusted. Good compliance to amblyopia therapy was associated with better BCVA (logMAR) at last follow-up (*β* = −0.40, 95% CI = −0.53 to −0.27, *p* < 0.0001) with laterality, opacity type, and extraction time adjusted.

**Conclusions:**

For Chinese infants with congenital cataract, an earlier primary congenital cataract surgery at an age of 3 to 15 months is associated with a better visual outcome. Good compliance to amblyopia therapy was also significant to visual outcome.

## 1. Introduction

Infantile congenital cataract is a major cause of lifelong visual impairment [1–4]. It is estimated that 200,000 children worldwide are blind from cataracts [5], and improving visual outcomes for affected children is a priority for the global vision 2020 initiative [6]. Presently, there are two main surgical procedures to treat pediatric cataracts: primary intraocular lens (IOL) implantation and secondary IOL implantation. Previous studies have reported that similar visual outcomes and complications may be observed in both surgeries [7]. However, an increasing number of surgeons still prefer aphakia as their initial management strategy in children younger than 1 year [8], considering subsequent eye growth and a myopic shift in children [9, 10]. Moreover, in children with associated microcornea, persistent fetal vasculature, rubella cataract, and so on, it may be preferable to leave them aphakic at the primary surgery for not increasing the risk of some complications, such as visual axis opacity and persistent hyperplastic primary vitreous [11].

Visual outcomes are largely dependent on the timing of the surgery. Congenital and infantile cataracts, if not treated promptly, lead to profound and irreversible vision loss. The extraction of congenital unilateral cataracts at 6–8 weeks of age along with optical correction and occlusion therapy may result in near-normal visual acuity [12–14]. Lambert et al. [15] reported a linear trend towards worse visual acuity outcomes at 4 to 6 years old with increasing age at surgery, but it was not statistically significant. Birch and Stager [13] reported that if surgery was delayed by 0–14 weeks, the mean visual acuity decreased by 1 line with each 3-week delay in surgery, and 14 weeks to 31 weeks, final visual acuity was independent of the subject's age at surgery, averaging 20/80.

These studies [12–15] all reported on primary IOL implantation. However, little is known about the relationship between time of primary cataract surgery and visual outcomes for secondary IOL implantation, which is more clinical. Additionally, it is generally thought that 6 weeks and 10 weeks may be the key cutoffs for cataract extraction for unilateral visual deprivation and bilateral visual deprivation, respectively [15–17]. However, in China, it is noted that large numbers of children undergo primary cataract surgery after 6 weeks of age. Thus, existing studies may not provide instructions for predicting visual rehabilitation. Finally, previous studies have reported that good compliance to amblyopia therapy was associated with a better visual outcome in children with congenital cataract, including the study from the Infant Aphakia Treatment Study [18, 19]. Thus, we also attempted to compare the independent effect between the compliance and the cataract extraction time. This study is a secondary case series analysis based on a published paper named “Long-Term Visual Outcomes of Secondary Intraocular Lens Implantation in Children with Congenital Cataracts” [20]. The original cohort is from a large-scale eye hospital in China, and it is a valuable clinical database including congenital cataract data from 7 years and follow-up for about 8 years. This study aimed to analyze the independent impact of cataract extraction time on visual rehabilitation for secondary IOL implantation and further study the relationship between the compliance to amblyopia therapy and the cataract extraction time by exploring their effects on visual outcomes.

## 2. Materials and Methods

### 2.1. Subjects and Methods

The overall design of this case series and the results of the visual acuity assessment have been published previously [20]. We retrospectively reviewed the records of all infants with congenital cataracts who underwent secondary IOL implantation in the Eye and ENT Hospital of Fudan University from January 1, 2001, to December 31, 2007, and the minimum follow-up inclusion was 5 years. Briefly, the preliminary aim for the case series was to assess the long-term visual outcomes and factors affecting visual results in children undergoing secondary intraocular lens implantation. Children with traumatic cataract, retinopathy of prematurity, congenital glaucoma, microphthalmos, persistent fetal vasculature, Marfan's syndrome, and other anterior or posterior segment anomalies were excluded. And children with systemic diseases (affecting learning ability) were also excluded. One eye was randomly selected in children with bilateral cataracts. The study was approved by the Ethics Committee of the Eye and ENT Hospital, Fudan University, Shanghai, China, and all patient information was collected after obtaining written informed consent from the children's guardians on their behalf. The consent procedure was also approved by the ethics committee and in accordance with the tenets of the Declaration of Helsinki.

### 2.2. Surgery

All children underwent manual anterior capsulorhexis, irrigation, and aspiration of cataracts, posterior capsulectomy, anterior vitrectomy, and the secondary postponed IOL implantations. After surgery, the operated eyes were treated with topical antibiotics, corticosteroids, and nonsteroidal anti-inflammatory drugs. All of the surgeries (including initial cataract extraction and secondary IOL implantation) were conducted by the same experienced doctor (Yi Lu), with the patients under general anesthesia.

### 2.3. Measures and Records

Laterality referred to the congenital cataracts in patients, which was unilateral or bilateral. The type of cataract was categorized according to opacity degree (partial or total). Cataracts that hindered vision of the red reflex during an ocular fundus examination were considered total. For both the initial and secondary surgeries, the patients were examined preoperatively and postoperatively at one day, one week, two weeks, one month, and then at six-month intervals until the last follow-up. All aphakic children after the cataract extraction were prescribed spectacles or contact lenses combined with management of amblyopia. Compliance to amblyopia therapy was reported as none, poor, or good if 0–25%, 25–75%, or 75–100%, respectively, of the prescribed hours were reported [20]. Best-corrected visual acuity (BCVA) was determined using a standard crowded Snellen chart and converted to the logarithm of the minimum angle of resolution (logMAR) for statistical analysis.

### 2.4. Analytic Methods

All analyses were performed using Empower (R) (http://www.empowerstats.com, X&Y Solutions Inc., Boston, MA) and R (http://www.R-project.org). Descriptive statistics (mean ± SD, median (min–max), *N* (%), etc.) were used to summarize baseline characteristics. Generalized linear regression analyses were performed after adjusting the covariates for analyzing the independent influence between extraction times and long-term visual outcomes. Survival curves were estimated according to the Kaplan-Meier method and compared with a log-rank test. Generalized linear regression analysis was also performed to assess the impact of compliance with amblyopia therapy to long-term visual outcomes. The important results were presented via *β* (95% CI) or OR (95% CI), and *p* value of less than 0.05 was considered statistically significant.

## 3. Results

From 2001 to 2007, 110 eyes underwent secondary IOL implantation surgery meeting the inclusion criteria, including 65 (59.1%) males and 45 (40.9%) females. The patients' demographics were listed in Table 1. Compliances to amblyopia therapy were defined as none, poor, and good, accounting for 24 (21.8%), 27 (24.5%), and 59 (53.6%), respectively. The cataract extraction time ranged from 3 months to 15 months. Median age at secondary IOL implantation was 35.0 (22.0–184.0) months. In addition, the mean follow-up time from IOL implantation was 99.3 ± 23.6 months, and the age at last follow-up was 12.6 ± 4.5 years. Median logMAR BCVA at last follow-up time was 0.70 (0.00–2.00).

**Figure 1:**
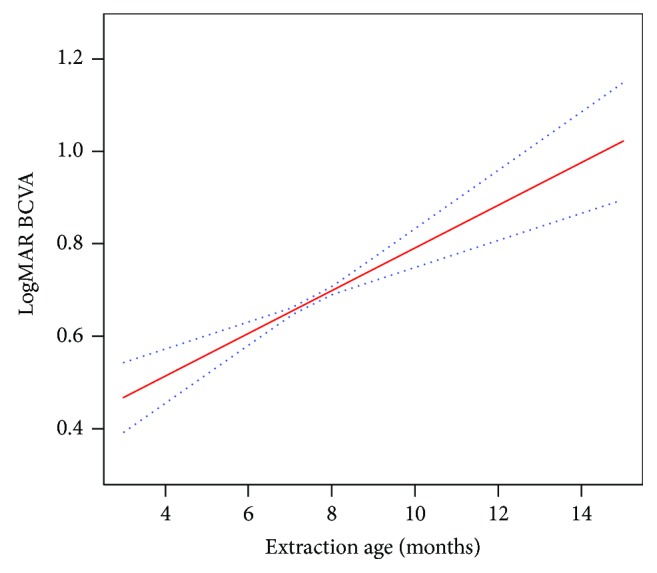
Smooth curve fitting of cataract extraction times and long-term visual outcomes. In the figure, the solid line indicates the estimated long-term visual outcomes, and the dotted lines represent the point wise 95% confidence interval. Laterality, opacity type, and compliance to amblyopia therapy have been adjusted.

Smooth curve fitting (Figure 1) was performed after the adjustment of relevant baseline variables, and the resultant curve showed a linear association of cataract extraction age with long-term visual acuity. The results of multivariate analysis suggested that extraction time remained significantly associated with long-term logMAR BCVA (*p* < 0.0001) (Table 2) after the adjustment of the confounding factors. Covariate screening was analyzed using computer software. Screening criteria I included risk factors producing > 10% change in the regression coefficient after introduction into the basic model, and screening criteria II included screening criteria I or *p* values of the regression coefficient to dependent variables that were less than 0.1. The results showed that the laterality and the compliance to amblyopia therapy met the filter criteria I and that the laterality, the compliance to amblyopia therapy, and the opacity type met criteria II. The regression coefficient in the nonadjusted model was 0.06 (95% CI = 0.04–0.08, *p* < 0.0001), and the regression coefficients of the adjust I and adjust II models were 0.05 (95% CI = 0.03–0.07, *p* < 0.0001) and 0.04 (95% CI = 0.03–0.06, *p* < 0.0001), respectively (Table 2). What is more, the interaction of laterality showed a regression coefficient of 0.04 (95% CI = 0.02–0.06, *p* = 0.0001) in bilateral and 0.05 (95% CI = 0.03–0.08, *p* < 0.0001) in unilateral (*P* = 0.2629), respectively, after adjusting for opacity type and compliance to amblyopia therapy.

**Figure 2:**
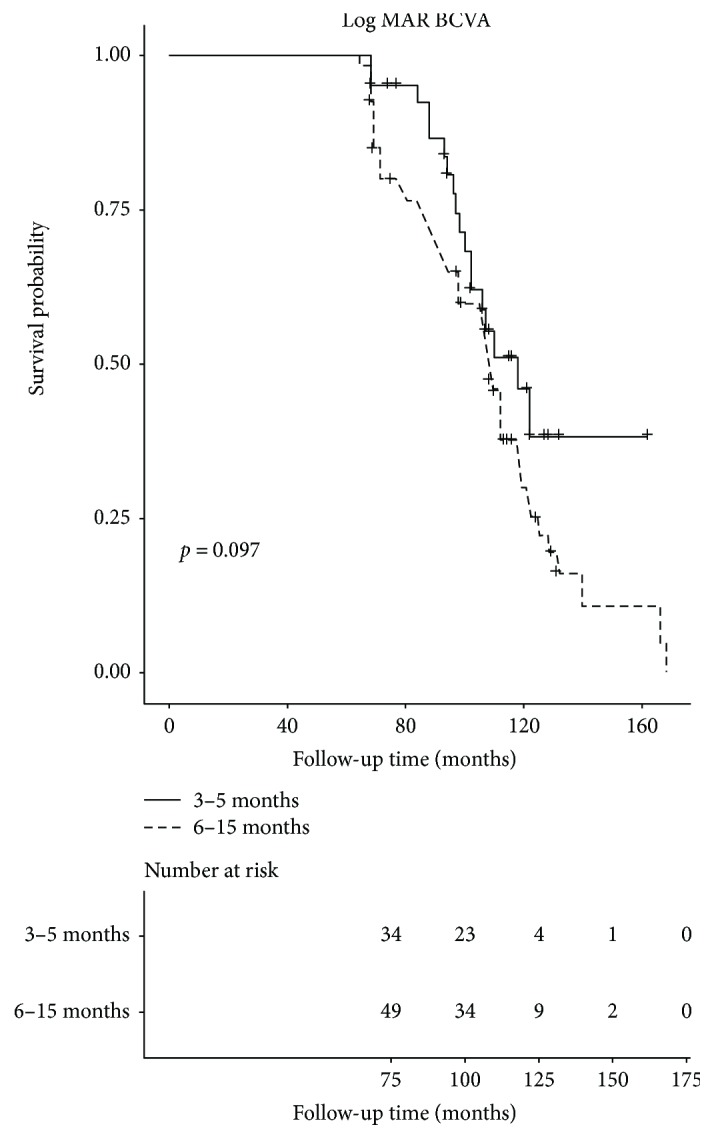
Kaplan–Meier analysis of the probability of good long-term visual acuity. Kaplan–Meier analysis showed the probability of good long-term visual acuity, which was defined as logMAR BCVA less than 0.4. The solid line indicates the patients who had lenses extracted at 3 months to 5 months, and the dotted lines represent those who had lenses extracted at 6 months to 15 months. (log rank, *p* = 0.097).

To assess the relationship between extraction times and visual outcomes rigorously, two groups were divided separately by the median. The cataract extraction age was divided into the low group (3.0–5.0 months) and high group (6.0–15.0 months), and the logMAR BCVA was grouped by 0.4. The odds ratio (OR) of extraction time groups in the nonadjusted model was 3.20 (95% CI = 1.43–7.15, *p* = 0.0045), and 3.05 (95% CI = 1.30–7.18, *p* = 0.0105) and 6.50 (95% CI = 2.02–20.89, *p* = 0.0017) in the adjust I and adjust II models, respectively (Table 2). Other regression coefficients and ORs are also shown in Table 2. The low-age group and bad logMAR BCVA group were the references. Meanwhile, Kaplan–Meier survival analysis was also shown in Figure 2 after stratified by the cataract extraction time (log-rank, *p* = 0.097).

Based on the same database, the association was also probed between compliance to amblyopia therapy and visual outcomes (Table 3). Multivariate regression analysis was used in the adjusted model, and the screening criteria included risk factors producing > 10% change in the regression coefficient after introduction into the basic model. Although there was no evidence indicating that the poor-group had a better long-term visual outcome when making the none-group as the reference, good compliance showed better visual acuity both in the nonadjusted model (−0.33, 95% CI = −0.55 to −0.12, *p* = 0.0033) and adjusted model (−0.40, 95% CI = −0.53 to −0.27, *p* < 0.0001).

## 4. Discussion

A published paper [20] showed the influences on visual outcomes in congenital cataract children undergoing secondary IOL implantation are laterality, cataract type, age at initial cataract extraction, compliance to amblyopia therapy, and refractive error. However, there was no interpretation about the regression coefficients (*β*), which is the core finding of the multiple regressions. To get a more scientific and clinically significant result, this study reanalyzed the rare cases and showed the independent impact of risk factors which could be intervened.

There were some studies which indicated that the time of surgery may have a high correlation with visual prognosis in primary IOL implantation [21–23], but few studies have focused on congenital cataract children who were left aphakic for years until IOL implantation was conducted. Previous studies indicated that the immature visual system is still reliant on subcortical pathways [24, 25] in the early neonatal period, and visual disturbance does not appear to influence final visual outcomes during this latent period. The generally recognized latent good visual period was 6 weeks in unilateral visual deprivation and 10 weeks in bilateral visual deprivation for primary IOL implantation [15–17]. Although the visual outcome after the 10-week extraction time was not as good as it was during this latent period, its rehabilitation was still crucial in clinic. In China, many congenital cataract children had surgery after 10 weeks, and the possible reasons may be the large number of patients and delayed presentation.

Smooth curve fitting with the adjustment of relative baseline variables showed a linear association of cataract extraction times and visual acuities at long-term follow-up (Figure 1). The regression coefficient in three regression models was approximately 0.05 (Table 2), which means that BCVA at the end of the follow-up may reduce 0.05 logMAR with each month's delay in primary cataract surgery (from 3 months to 15 months). In other words, if children experienced a delayed operation for one year, the logMAR BCVA after about 8 years may add 6 lines on the ETDRS charts. This requires the attention of both surgeons and parents. Further, the odds of worse visual outcomes, that is, logMAR visual acuity > 0.4 (OR 6.5 after making the adjustment II) in children in the delayed cataract surgery group was 5.5-fold higher than in children in the early surgery group. Although the *p* value (0.097) in the Kaplan–Meier analysis (Figure 2) was not statistical significant, we can still find that the low age group may have a higher survival probability. And actually the *p* value was 0.031 (*p* < 0.05) when we reperformed the analysis as the logMAR BCVA was grouped by 1.0 to reach a more sensitive result. This difference may be due to the sample size, but we believe that difference is still significant for surgeons and parents.

It was reported that the latent period for good visual outcomes was 6 weeks in unilateral visual deprivation and 10 weeks in bilateral visual deprivation when conducting lens extraction and IOL implantation at the same time [15–17]. In this study, there was no evidence which indicated that laterality had an interaction with time of primary cataract surgery and visual acuity (*p* = 0.2629) when primary cataract surgery was conducted at 3 months to 15 months of age while patients remained aphakic for years. However, the regression coefficient of bilateral cases (0.04, 95% CI = 0.02–0.06, *p* = 0.0001) and unilateral cases (0.05, 95% CI = 0.03–0.08, *p* < 0.0001) still indicated that bilateral patients may have better prognosis with time going by. Many animal studies have demonstrated that bilateral visual deprivation affects the visual cortex differently than unilateral visual deprivation during the sensitive period [26, 27]. Lambert et al. [15] had also revealed in their study that children with dense bilateral congenital cataracts had a different latent period comparing with unilateral congenital cataracts. However, the time of primary cataract surgery in this study is well beyond the latent period. That may cause the results to be not sensitive to achieve the statistical significance. Additionally, the significance may also be attributed to the relatively small sample size of unilateral patients in this study, and it still needed further investigation.

The other modifiable factor in this database was the compliance to amblyopia therapy. Congenital cataracts happen in the core period of visual development, and amblyopia therapy is inevitable except for the operation. An infant with a unilateral congenital cataract will need long-term “aggressive” occlusion therapy (6 h to 8 h daily) if useful visual function is to be achieved in the affected eye [13]. Drews-Botsch et al. [18] illustrated the association of occlusion throughout the preschool years with improved visual acuity using the date from Infant Aphakia Treatment Study (IATS). The importance of occlusion therapy was consistent with most previous assessments of vision rehabilitation in children from IATS [19, 28]. In this study, good compliance resulted in better visual acuity in the adjustment model (−0.40, 95% CI = −0.53 to −0.27, *p* < 0.0001) than no compliance, which means that good occlusion therapy would enhance the BCVA approximately by 0.4 logMAR. What is more interesting, this enhancement is equivalent to an advanced operation at about 10 months (the regression coefficient of extraction time was 0.04 per month in this study). For example, if a congenital cataract infant comes to see the doctor at 13 months of age, he can achieve the final visual acuity as if he had undergone the surgery at 3 months of age, provided there is good compliance to amblyopia therapy. This was really significant for the patients who had missed the optimal operation time.

There are limitations in this study. The selection and explanation of the baseline data are unchangeable because it was based on a published database. Additionally, the tools and the method to record the compliance to amblyopia therapy were not described clearly. Efforts were made to get in touch with the corresponding author, but it did not work. In China, the traditional patching regimens were in accordance with the views of von Noorden that 1-year-old children should take the 3 : 1 rule patching, appropriately extending the patching time of the dominant eye with the increasing age [29, 30]. We believe that improved patching regimens may be associated with a better visual outcome than traditional patching regimens, however, provided that parents and surgeons recognize the significance of the compliance. Finally, although the quantity of samples in this study was larger than most previous surveys, further studies are still needed for the interactions, such as laterality.

## 5. Conclusions

In summary, an earlier primary congenital cataract surgery at 3 to 15 months of age in Chinese infants is associated with better visual outcomes. These data might be used to encourage surgeons and parents to conduct the primary cataract surgery as soon as possible, even if they had missed the latent good visual outcome period. Also, compliance to amblyopia therapy is an important modifiable risk factor that may help negate the effect of delayed surgery. However, further studies are needed to confirm this hypothesis.

## Figures and Tables

**Table 1:** Children's demographics.

Characteristics	*N* = 110
Sex	
Male	65 (59.1%)
Female	45 (40.9%)
Laterality	
Bilateral	76 (69.1%)
Unilateral	34 (30.9%)
Opacity type	
Partial opacity	46 (41.8%)
Total opacity	64 (58.2%)
Compliance to amblyopia therapy	
None	24 (21.8%)
Poor	27 (24.5%)
Good	59 (53.6%)
Median (min–max), age at cataract extraction, months	7.5 (3.0–15.0)
Age at cataract extraction	
Median (min–max), low, months	4.0 (3.0–5.0)
Median (min–max), high, months	9.0 (6.0–15.0)
Median (min–max), age at IOL implantation, months	35.0 (22.0–184.0)
Mean (SD), follow-up time from IOL implantation, months	99.3 (23.6)
Mean (SD), age at last follow-up, years	12.6 (4.5)
Median (min–max), UCVA	0.12 (0.01–1.00)
Median (min–max), BCVA	0.20 (0.01–1.00)
Median (min–max), logMAR BCVA	0.70 (0.00–2.00)

UCVA: uncorrected visual acuity; BCVA: best-corrected visual acuity.

**Table 2:** Multivariate regression analysis of cataract extraction times and long-term visual outcomes.

	Nonadjusted	Adjust I	Adjust II
	LogMAR BCVA [*β* (95% CI), *p* value]		
Age	0.06 (0.04, 0.08), <0.0001	0.05 (0.03, 0.07), <0.0001	0.04 (0.03, 0.06), <0.0001
Age (high versus low)^a^	0.29 (0.12, 0.47), 0.0014	0.23 (0.09, 0.37), 0.0005	0.22 (0.10, 0.34), 0.0005
	Bad logMAR BCVA^b^ [OR (95% CI), *p* value]		
Age	1.35 (1.16, 1.57), 0.0001	1.35 (1.15, 1.58), 0.0002	1.62 (1.26, 2.09), 0.0002
Age (high versus low)^a^	3.20 (1.43, 7.15), 0.0045	3.05 (1.30, 7.18), 0.0105	6.50 (2.02, 20.89), 0.0017

^a^The high extraction age was 4.0 (3.0–5.0) months [median (min–max)] and the low extraction age was 9.0 (6.0–15.0) months [median (min–max)]; ^b^the bad visual acuity was BCVA equal or worse than 20/50; nonadjusted model adjust for: none; adjust I model adjust for: laterality; compliance to amblyopia therapy; adjust II model adjust for: laterality; opacity type; compliance to amblyopia therapy.

**Table 3:** Multivariate regression analysis of compliance with amblyopia therapy and long-term visual outcomes.

	LogMAR BCVA [*β* (95%CI), *p* value]	
Amblyopia therapy compliance	Nonadjusted	Adjusted
None^a^	Reference	Reference
Poor^b^	−0.12 (−0.37, 0.13), 0.3536	−0.10 (−0.25, 0.05), 0.2012
Good^c^	−0.33 (−0.55, −0.12), 0.0033	−0.40 (−0.53, −0.27), <0.0001

^a,b,c^Compliance with amblyopia therapy was reported as none, poor, or good if 0–25%, 25–75%, or 75–100%, respectively, of the prescribed hours were reported; nonadjusted model adjust for: none; adjust model adjust for: laterality; opacity type; extraction age.
